# Hybrid Amyloid Quantum Dot Nano-Bio Assemblies to Probe Neuroinflammatory Damage

**DOI:** 10.1021/acschemneuro.4c00183

**Published:** 2024-08-15

**Authors:** Wesley Chiang, Jennifer M. Urban, Francine Yanchik-Slade, Angela Stout, Jennetta M. Hammond, Bradley L. Nilsson, Harris A. Gelbard, Todd D. Krauss

**Affiliations:** † Department of Chemistry, University of Rochester, Rochester, New York 14627-0216, United States; ‡ The Institute of Optics, University of Rochester Medical Center, Rochester, New York 14627-0216, United States; § Department of Biochemistry and Biophysics, University of Rochester Medical Center, Rochester, New York 14642, United States; ∥ Center for Neurotherapeutics Discovery and Department of Neurology, University of Rochester Medical Center, Rochester, New York 14642, United States; ⊥ Departments of Pediatrics, Neuroscience, and Microbiology and Immunology, University of Rochester Medical Center, Rochester, New York 14642, United States

**Keywords:** quantum dots, neuronal imaging, biomimetic, neurotoxic oligomers, fluorescence microscopy, amyloid, Alzheimer’s

## Abstract

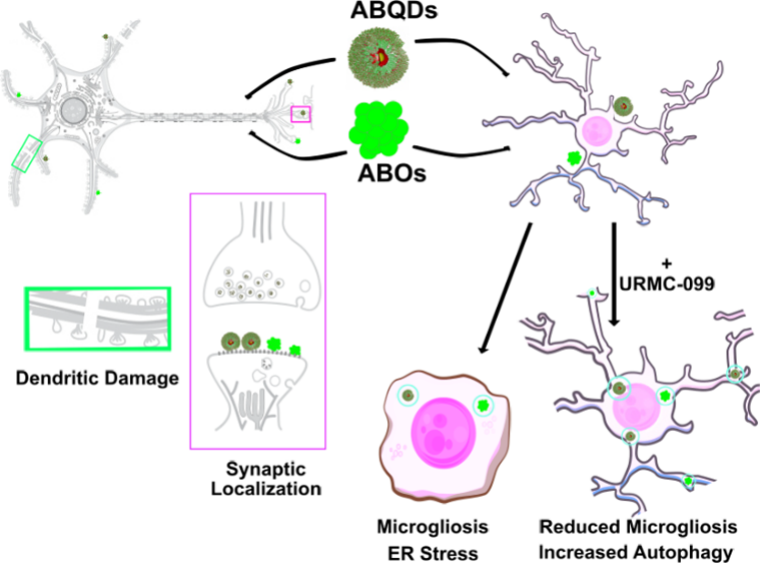

Various oligomeric species of amyloid-beta have been proposed to play different immunogenic roles in the cellular pathology of Alzheimer’s Disease. The dynamic interconversion between various amyloid oligomers and fibrillar assemblies makes it difficult to elucidate the role each potential aggregation state may play in driving neuroinflammatory and neurodegenerative pathology. The ability to identify the amyloid species that are key and essential drivers of these pathological hallmarks of Alzheimer’s Disease is of fundamental importance for also understanding downstream events including tauopathies that mediate neuroinflammation with neurologic deficits. Here, we report the design and construction of a quantum dot mimetic for larger spherical oligomeric amyloid species as an “endogenously” fluorescent proxy for this cytotoxic assembly of amyloid to investigate its role in inducing inflammatory and stress response states in neuronal and glial cell types. The design parameters and construction protocol developed here may be adapted for developing quantum dot nano-bio assemblies for other biological systems of interest, particularly neurodegenerative diseases involving other protein aggregates.

Despite decades of research into the underlying mechanisms that give rise to Alzheimer’s Disease (AD), no clear consensus has emerged as to which cellular phenomenon–amyloidosis, tauopathy, inflammation, oxidative stress–truly drives cognitive impairment.^2^ Based on neuropathologic and genetic evidence,^3^ deposition of amyloid-β (Aβ) peptide in the brain gives rise to the amyloid cascade hypothesis in which various oligomeric species of Aβ, specifically spherical aggregates,^4−6^ have been implicated as being drivers of neurotoxicity.^7−10^ This hypothesis has heavily influenced development of therapeutic interventions, many of which have targeted metabolism and antibody-mediated removal, with antibodies demonstrating varying degrees of efficacy in Aβ plaque removal;^3^ yet even the most promising of such antibody therapies have limited clinical efficacy.^11^

This, along with a lack of a strong correlate between decreasing amyloid burden and reduction of cognitive impairment,^12^ is not simply resolved by targeting other molecular drivers, such as tau.^13,14^ Rather, current evidence supports a hypothesis that Aβ may initiate tau pathology, and the associated burden of each drives inflammation at different stages within AD progression to mediate cognitive decline, rather than amyloid or tau being the sole malefactor.^2,15−20^ Thus, understanding the mechanisms by which Aβ can initiate progressive tauopathies and associated inflammatory dysregulation will aid in the development of future therapies.

While spheroidal amyloid oligomers are theorized to be the most likely amyloid species to be initiating neurotoxicity and neuroinflammation,^4−6^ mechanistic studies of how these oligomers may initiate signaling cascades that lead to dysregulated tau phosphorylation and misfolding are hampered by the dynamic interconversion of amyloid species among one another. The resultant assemblies can range from spherical and spheroidal oligomers, to protofibrils, much larger fibrillar assemblies and plaques;^1,8,10^ a mixture of some or all these species may exist at any time and thereby limit the ability to delineate the individual roles each aggregate state may play.

It is for these reasons that we have used the following design of a quantum dot biomimetic nano-bio assembly for spheroidal Aβ oligomers (ABOs), the most commonly implicated neurotoxic species of Aβ,^4−6^ as a tool to interrogate multiple pathways for aberrant neuroimmune signaling and by extension, in future studies, using a similarly constructed tau biomimetic to investigate spreading tauopathies. These nano-bio tools have the advantage of allowing us to study in situ signaling at the cellular and subcellular level, with the potential to study nanoscale events that may reveal further clues as to how Aβ can initiate spreading tauopathies.

However, a well-known potential disadvantage of using QDs as biological imaging probes is that they often have drastically different physical characteristics (i.e., size, valency, target density, etc.) compared to the biological target molecule they are labeling.^21,22^ Indeed, functionalization of QDs with biomolecules generally results in the formation of large, spherical probes presenting many copies of the biomolecule of interest in the QD surface.^23,24^ Based on differences in size and ligand valency, it is reasonable to assume that QDs may behave differently than the ligand alone in a biological context.^21,22,25^

When using QD probes it is common to focus on overall cytotoxicity or biodistribution of QDs in vitro and in vivo.^26−32^ However, it is far less common to investigate whether the biological system behaves appropriately in response to the biomolecular probe that is tethered to the surface of the QD.^33^ Specifically, the size and valency of ligand-functionalized QDs can be tailored to mimic endogenous spherical biological macromolecules, which can range from spherical oligomeric proteins to virus-like nanoparticles to even lipid vesicles.^33^ As such, we posit that coating the QD surface with moieties of organic matter to produce a hybrid nano-bio construct resembling aggregated macromolecules, such as AβOs, a QD-based biomimetic may be constructed to be structurally and functionally recognized by cells akin to endogenous spheroidal AβOs (Figures 1 and S4). These spheroidal ΑβO-mimicking QDs (ABQDs) may serve as nanoscale biophysical tools to determine the mechanistic role of spherical AβOs in mediating neuroinflammation and subsequent tauopathies in AD pathology.

**Figure 1:**
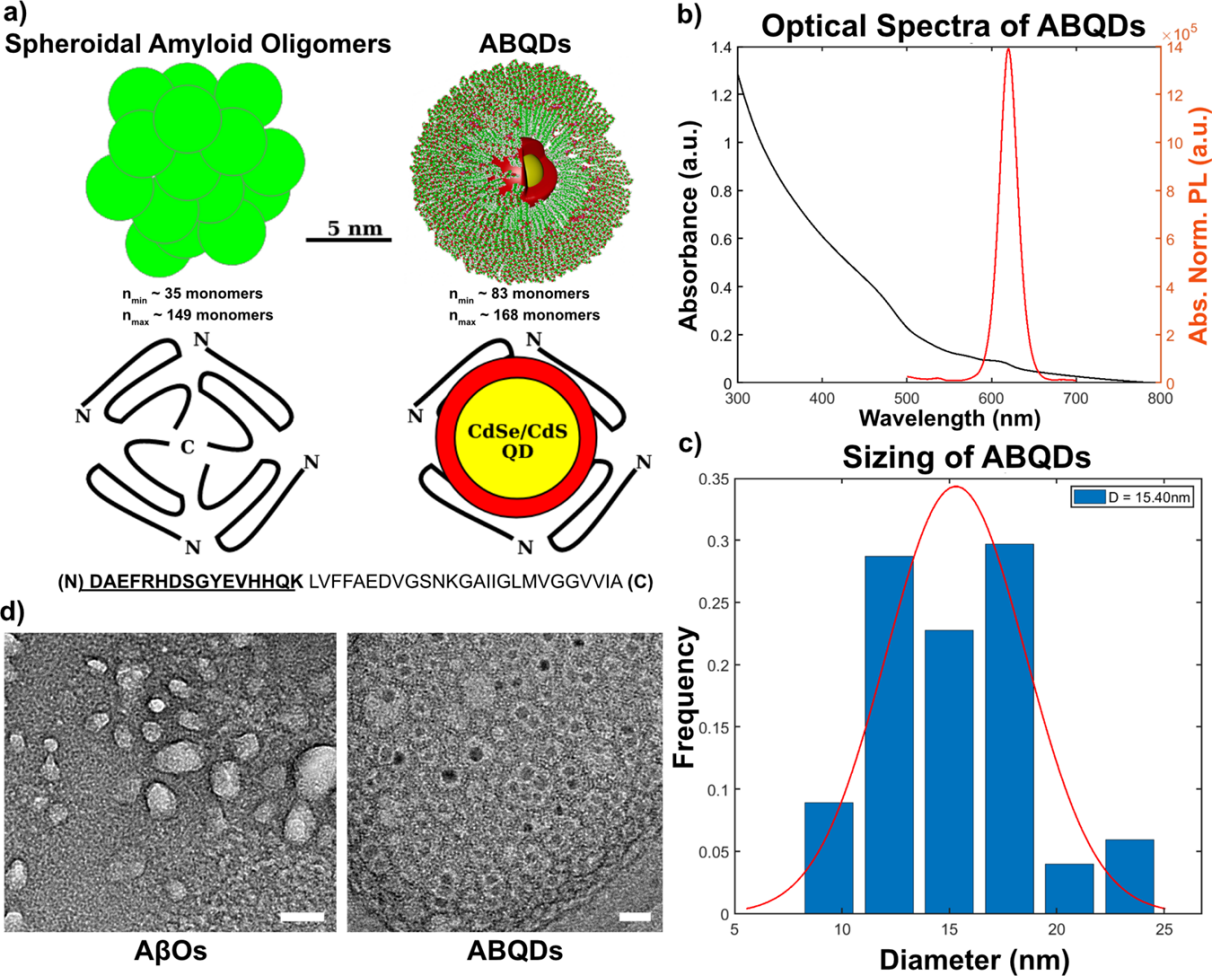
Diagram of conceptual framework behind ABQD design and characterization of optical and size properties. (a) Size comparison of spherical aggregates of amyloid-beta 42 peptides to a CdSe/CdS QD encapsulated in a DSPE-PEG_2k_ micelle (top), proposed oligomerization of peptides by Ahmed et al. compared to structural design of ABQDs (middle), where the first 16 amino acids of the N-terminus are synthesized (below) and used to functionalize the micellar surface.^1^ (b) Confirmation that ABQDs retain the optical properties of the original CdSe/CdS QDs. The absorbance exhibits low signal past the lowest energy transition of the QD (>620 nm) that arises from scattering from empty micelles. (c) DLS characterization of ABQD size distribution, with average hydrodynamic radius ≈ 15.4 nm. (d) TEM comparison of commercial spherical amyloid oligomers (AβOs; StressMarq, SPR-488) to constructed ABQDs. Negative staining with uranyl acetate produces lighter halo regions representing oligomers or micells. Dark puncta in lighter micelles are the QDs. Scale 25 nm.

Herein, we report the construction of ABQDs via micelle encapsulation of CdSe/CdS QDs in polymerized phospholipids decorated with 16-residue peptide sequences of Aβ(1–16). Functional mimicry of the ABQD nano-bio assemblies was assessed by measuring inflammatory hallmarks (i.e., dendritic beading, synaptic pruning, and astrogliosis)^34−40^ and calcium signaling in primary cultures of hippocampal neuroglia isolated from Sprague–Dawley rats. We also observed changes in phagocytic activity and activation of pro-inflammatory and endoplasmic reticulum (ER) stress responses in an immortalized microglial (BV-2) cell line. Finally, we show that these effects are attenuated by cotreatment with a broad-spectrum mixed-lineage kinase inhibitor, URMC-099, previously demonstrated to ameliorate these pathologic hallmarks with in vitro and in vivo models of AD.^41,42^ Taken together, our results highlight the development of a new, robust fluorescent nano-bio tool to mimic spherical aggregates of Aβ42 that can be utilized to elucidate neurotoxic mechanisms related to AD and the effectiveness of therapeutic interventions in mitigating these effects.

## Results and Discussion

### ABQDs Structurally Resemble Spheroidal AβOs

Our proposed nano-bio construct models the assembly of Aβ42 oligomers such that the N-terminal amino acid residues are exposed to the biological environment, serving as the antigenic region responsible for biomolecular recognition and signal transduction.^4−6^ Thus, we synthesized, via solid-phase peptide synthesis, the first 16 amino acids from the N-terminus of Aβ42 and attached the peptides to a phospholipid-PEG construct (DSPE-PEG_2k_) via a cysteine-maleimide conjugation scheme (Scheme S1). The successful synthesis of the peptide Aβ(1–16)-PEG-CG was validated using matrix-assisted laser desorption ionization time-of-flight (MALDI-TOF) mass spectrometry to confirm the presence of the expected mass product (Figures S1 and S3). Concentration curves were calibrated using an analytical high performance liquid chromatography (HPLC) and used to determine the concentrations of all syntheses of Aβ(1–16)-PEG-CG used in these experiments. Successful conjugation of the peptide with the lipid-PEG (DSPE-PEG_2k_-Aβ) was confirmed via analytical HPLC and MALDI-TOF (Figure S3) to show a shift in the elution time of the newly formed product (from 10.3 to 12.7 min) and associated mass expected (∼4300 Da) from the addition of the peptide (2279 Da) and the lipid-PEG (∼2000 Da). The broad distribution of mass peaks arises from the inherent polydispersity of polymer synthesis, however the centering of the peak and its distribution shifts in exact accordance with the well-defined, sharp peak of the peptide. This mass distribution is also reflected in the delayed HPLC elution time of the DSPE-PEG_2k_-Aβ(1–16) product, which broadens compared to the earlier, sharp elution peak of just the peptide.

However, beyond ensuring that the approach to decorate the QD surface appropriately modeled the exposed region of spheroidal AβOs, detailed characterization of the structural resemblance of ABQDs to endogenous Aβ42 spheroidal oligomers, as diagrammed in Figure 1a, is key to ensuring that the ABQDs can be used as a functional proxy. Thus, after micellar encapsulation of CdSe/CdS with DSPE-PEG_2k_-Aβ to form ABQDs, the heterogeneous population of mimetic constructs were separated into unique size fractions. Each size fraction was measured with dynamic light scattering (DLS) to identify the fraction that best fit the reported diameter of neurotoxic spheroidal amyloid oligomers (approximately 12–20 nm).^1,4,5,8^ We characterized absorbance and photoluminescence spectra of the ABQDs to ensure that desirable optical properties of QDs are retained (Figure 1b). This is complemented by the DLS data, in Figure 1c, and TEM micrographs (Figure 1d) showing a size distribution of 10–22.5 nm and rounded morphology similar to native ABOs. Thus, the isolated size fraction of interest contains CdSe/CdS encapsulated Aβ(1–16) functionalized micelles that structurally resemble spheroidal AβOs. Specifically, structural studies via solid-state nuclear magnetic resonance of spheroidal amyloid structures of a similar size range purportedly assemble where the C-terminal domains of monomers interact into structured globular domains while the 16 N-terminal amino acids are flexible and exposed on the outer region of these aggregates.^1,5,6^

### ABQDs are Immunoreactive to Common Amyloid Antibodies

**Figure 2:**
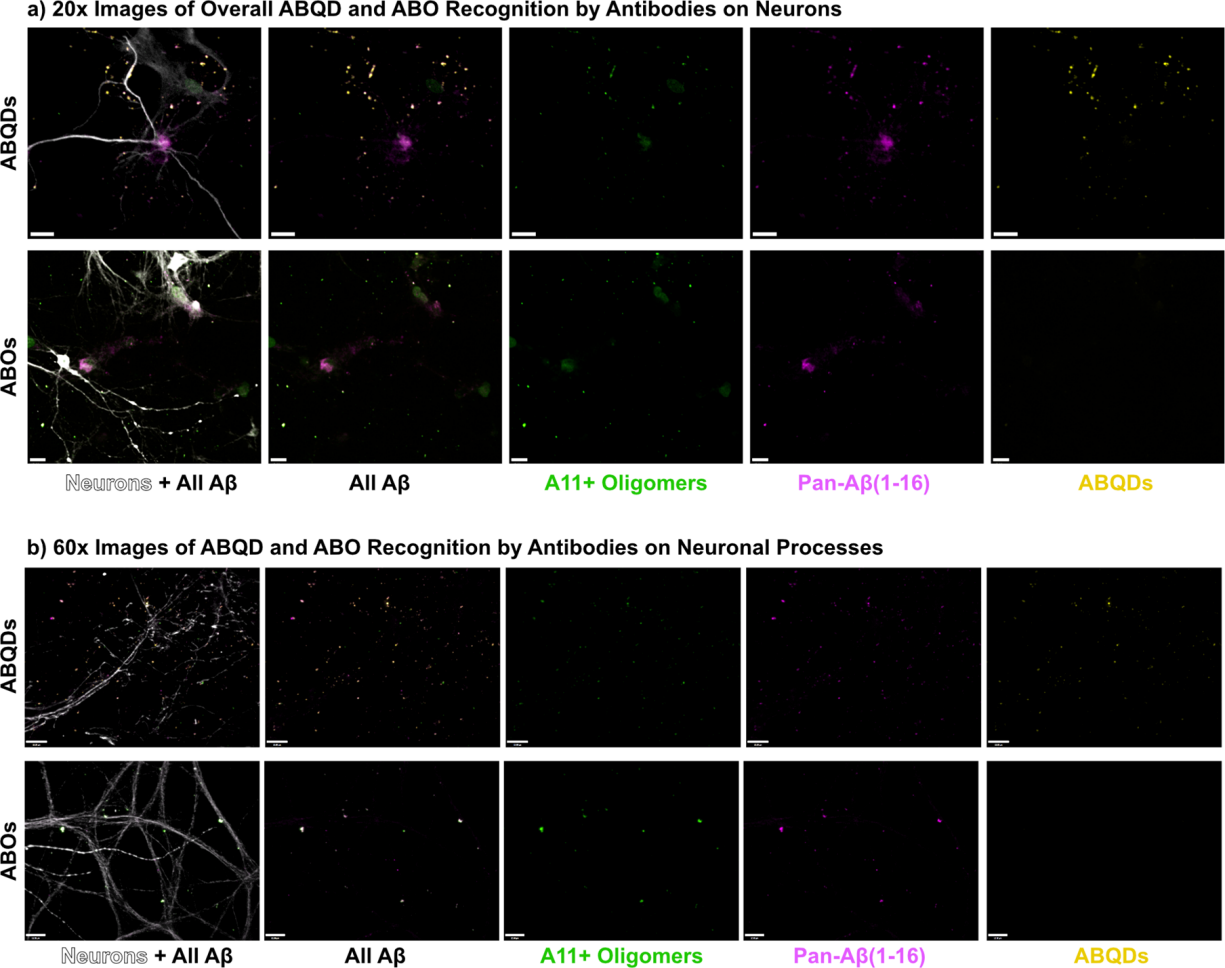
Validation of ABQD mimicry of spheroidal ABOs via immunoreactivity with known amyloid antibodies A11 and 6E10. Primary rat hippocampal neuroglial cultures were treated with ABQDs and ABOs, the fixed and immunostained with oligomeric conformationally selective A11 antibodies and pan-Aβ42 clone 6E10 antibody that targets the N-terminal Αβ(1–16) amino acids. (a) At 20× magnification ABQDs were confirmed to bind MAP2 positive neurons with similar selectivity as ABOs, with immunoreactivity to both A11 and 6E10 antibodies. (b) Further examination at 60× along neuronal processes demonstrated a neuroinflammatory hallmark, known as dendritic beading, that correlated with the binding of ABQDs and ABOs that were both immunoreactive to A11 and 6E10. Scale bars are 20 and 12 μm, respectively.

To confirm that ABQDs structurally mimicked naturally occurring spheroidal ABOs in a functional manner that could drive disease, we examined whether conventional amyloid antibodies would be immunoreactive and recognize the ABQDs. As shown in Figure 2, primary rat neuroglial cultures were treated with either ABQDs or commercially purchased ABOs and labeled for immunofluorescent imaging using an oligomer specific antibody (A11),^43^ a pan-amyloid antibody that recognizes the 16 N-terminal residues (6E10),^44^ and a neuronal marker (MAP2). Upon initial examination under 20× magnification (Figure 2a), we found strong colocalization of ABQDs and ABOs with neuronal processes. Indeed, amyloid targeting of neuronal surface proteins, particularly at synaptic densities,^45^ is a well-established phenomenon. Conversely, in control samples not treated with either ABQDs nor ABOs (Figure S5), no immunoreactivity to either A11 nor 6E10 was observed.

Importantly, both the A11 and 6E10 antibodies were immunoreactive for the both the ABOs and ABQDs, indicating that not only do the ABQDs properly display the N-terminal Aβ(1–16) residues on its surface (6E10 immunoreactivity), but also the morphology of the nano-bio assembly adequately mimics that of naturally occurring spherical ABOs (A11 immunoreactivity). This implies that the conformational presentation of surface residues and overall charge profile of the ABQDs are similar to that of other amyloidogenic oligomeric species, given that the A11 antibody is pan-oligomer marker for other neurotoxic spherical aggregates, such as synuclein, via conformational selectivity.^43^ Thus, in aggregate with the results of DLS, TEM, and MALDI-TOF, the immunoreactivity of ABQDs with both A11 and 6E10 antibodies on neurons is indicative of the proper structural and functional presentation of amyloid residues on its surface to adequately mimic spheroidal ABOs.

### ABQDs Recapitulate Aβ42-Associated Damage Phenotypes in Neurons and Astrocytes

**Figure 3:**
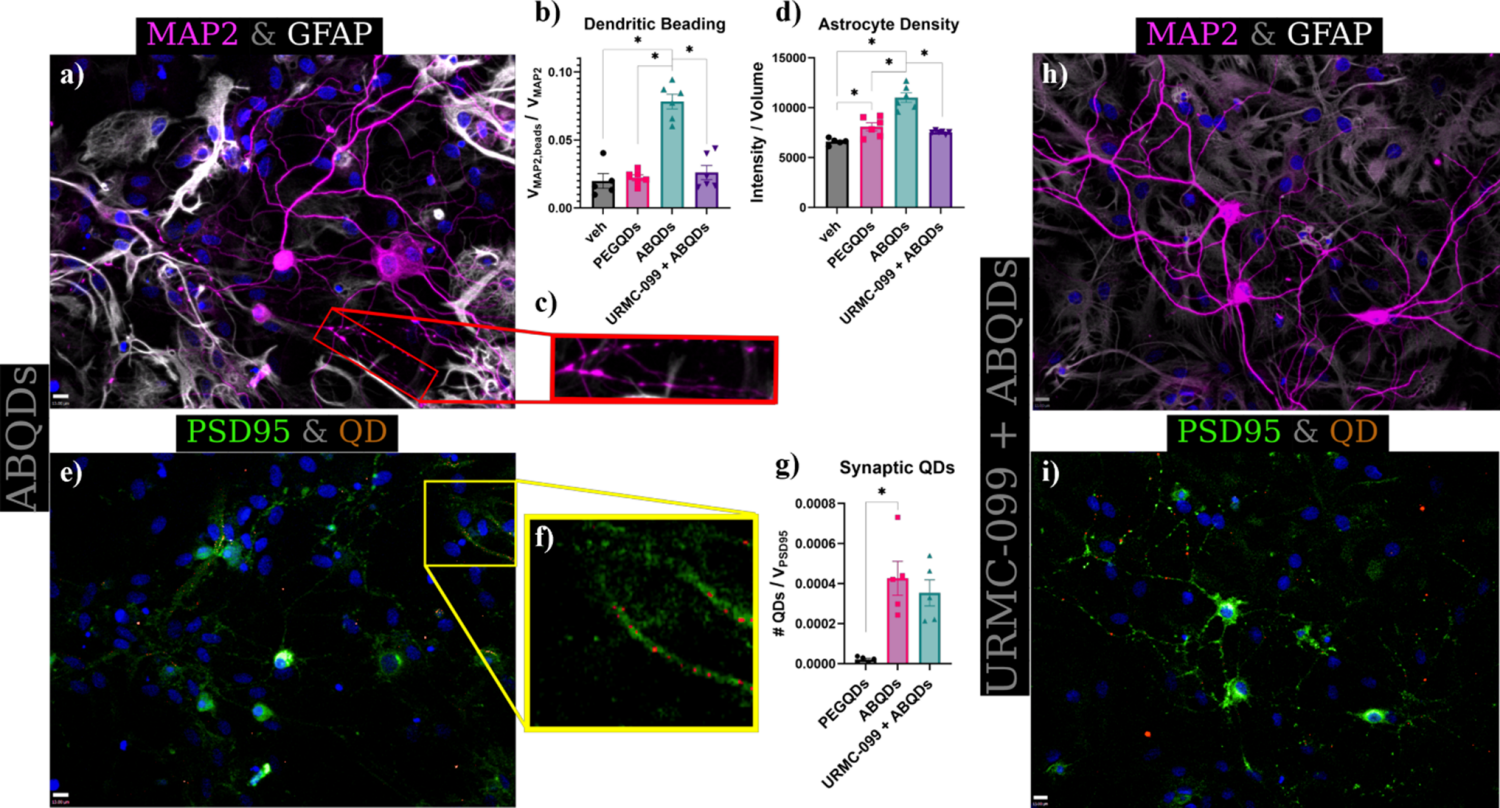
Neuroinflammatory activation by 50 nM of ABQDs that is mitigated by cotreatment with 100 nM of URMC-099. Immunofluorescent labeling of MAP2 (magenta) and GFAP (white) demonstrate the formation of focal swellings along neuronal processes (i.e., dendritic beading) complemented by astrogliosis in ABQD only treated neuroglial cultures (a,c); cotreatment with URMC-099 reduces the observation of such inflammatory hallmarks (h). In both treatment groups, ABQDs (orange) are shown to selectively colocalize with PSD95 (green), indicative of synaptic targeting and recognition commonly associated with amyloid pathology (e,f,i). Statistical analyses of these observations are performed using one-way ANOVA with a Holm-Sidak posthoc correction (*; *p* < 0.05) as plotted in (b,d,g). Nuclei are labeled by DAPI (blue). Scale bars are 13 μm.

After validating that the ABQDs have been constructed to mimic key structural parameters (size, general shape, and surface amino acid sequence) of endogenous spheroidal AβOs, we exposed primary cultures of rat hippocampal neurons and astrocytes to 50 nM of ABQDs. During examination of immunoreactivity for ABQDs to conventional amyloid antibodies, it became apparent that the presence of ABQDs were predominantly along neuronal processes and induced a common hallmark of neuronal damage known as dendritic beading (Figure 2b).^34^ These beaded discontinuities along the neuronal processes were also noted in the ABO treated samples. As such, we wanted to further investigate how well the ABQDs could recapitulate neuroinflammatory stress in primary rat neuroglial cultures in an amyloid-like pathology. Using immunocytochemical labeling of neuronal dendrites with microtubule-associated protein 2 (MAP2; magenta) and astrocytes with glial fibrillary acidic protein (GFAP; white), (Figure 3a), we were able characterize the functional capacity of the ABQDs to induce the presence of neuroinflammatory hallmarks, such as dendritic beading (Figure 3b, c) and astrogliosis (Figure 3d). Dendritic beading, defined as the observation of focal swellings in postsynaptic neuronal processes (Figure 3c), is a hallmark of synaptic injury and neurite damage commonly observed in AD neuropathology.^34−37,39^ Additionally, astrogliosis arises from the activation of a reactive, pro-inflammatory state in astrocytes typified by increased spatial distribution and expression levels of astrocytic cytoskeletal components, such as GFAP.^40,46,47^

While astrogliosis is a general hallmark of neuroinflammation, direct modulation of astrocyte activation and phenotype has been implicated in toxic AβO function.^40,48,49^ Comparatively, treating neuroglial cultures with 50 nM of micelle encapsulated quantum dots lacking Aβ(1–16) functionalization (PEGQDs, Figure S6), results in little or no dendritic beading compared to vehicle treated groups (Figure 3b), but a small, yet significant, induction of astrogliosis (Figure 3d). We attribute this discrepancy in the activation of inflammatory profiles in neurons versus astrocytes to arise from the recognition of PEGQDs by astrocytes as nonspecific extracellular debris that results in phagocytosis or endocytosis of PEGQDs by astrocytes (Figure S7). This associated astrogliosis may be seen as homeostatic regulation of the neuronal environment.^38,50−52^ However, astrogliosis is likely exacerbated by an amyloid specific interaction with the ABQDs, potentially mediated through complement dependent recognition of Aβ at synapses.^40,49,53−58^ Furthermore, progressive accumulation of Aβ and tau in 5X-FAD and JNPL3 mice respectively can induce reductions in PSD-95 in apical hippocampal dendrites, with a similar phenomenon in hippocampal AD brain sections.^45^ When examining the association of ABQDs compared to PEGQDs to neurons, we see that without the functionalization of Aβ(1–16) on the micellar surface, PEGQDs are unable to bind to postsynaptic densities (PSD95; green), while ABQDs exhibit specific association predominantly to PSD95 of neurons in the cultures (Figure 3e–g). Therefore, the neuronal damage mediated by the ABQDs is due to an amyloid-dependent biomolecular interaction with the neurons.

### URMC-099 Attenuates ABQD Induced Inflammation

Previously, we have shown that a small-molecule kinase inhibitor against mixed-lineage kinases (MLKs), known as URMC-099, acted in an anti-inflammatory and neuroprotective manner in murine models of various neuroinflammatory and neurodegenerative disorders.^41,59−63^ While most of these studies have been conducted in either murine models, or in cultures focused more on microglia, URMC-099 may also elicit protective effects directly in neurons and astrocytes, given the ubiquitous expression and subsequent role of MLKs in nearly all eukaryotic cell types.^64^ As such, we cotreated the neuroglial cultures with both 50 nM ABQDs and 100 nM URMC-099 to see if a direct inhibition of pro-inflammatory kinase signaling in neurons and astrocytes would reduce the manifestation of neuroinflammatory hallmarks. In agreement with our hypothesis of direct inhibition of MLKs, we saw a reduction in the amount of dendritic beading and astrogliosis in the cotreated cultures (Figure 3b,d,h). This neuroprotection was not due to URMC-099 mediated disruption of ABQD recognition of synaptic targets, given that we observed no significant (one-way ANOVA + Holm-Sidak posthoc; *p* > 0.05) difference in the fraction of ABQDs colocalized with PSD-95 in either treatment groups (Figure 3g,i).

### ABQDs Induce Changes in Neuronal Calcium Transients

**Figure 4:**
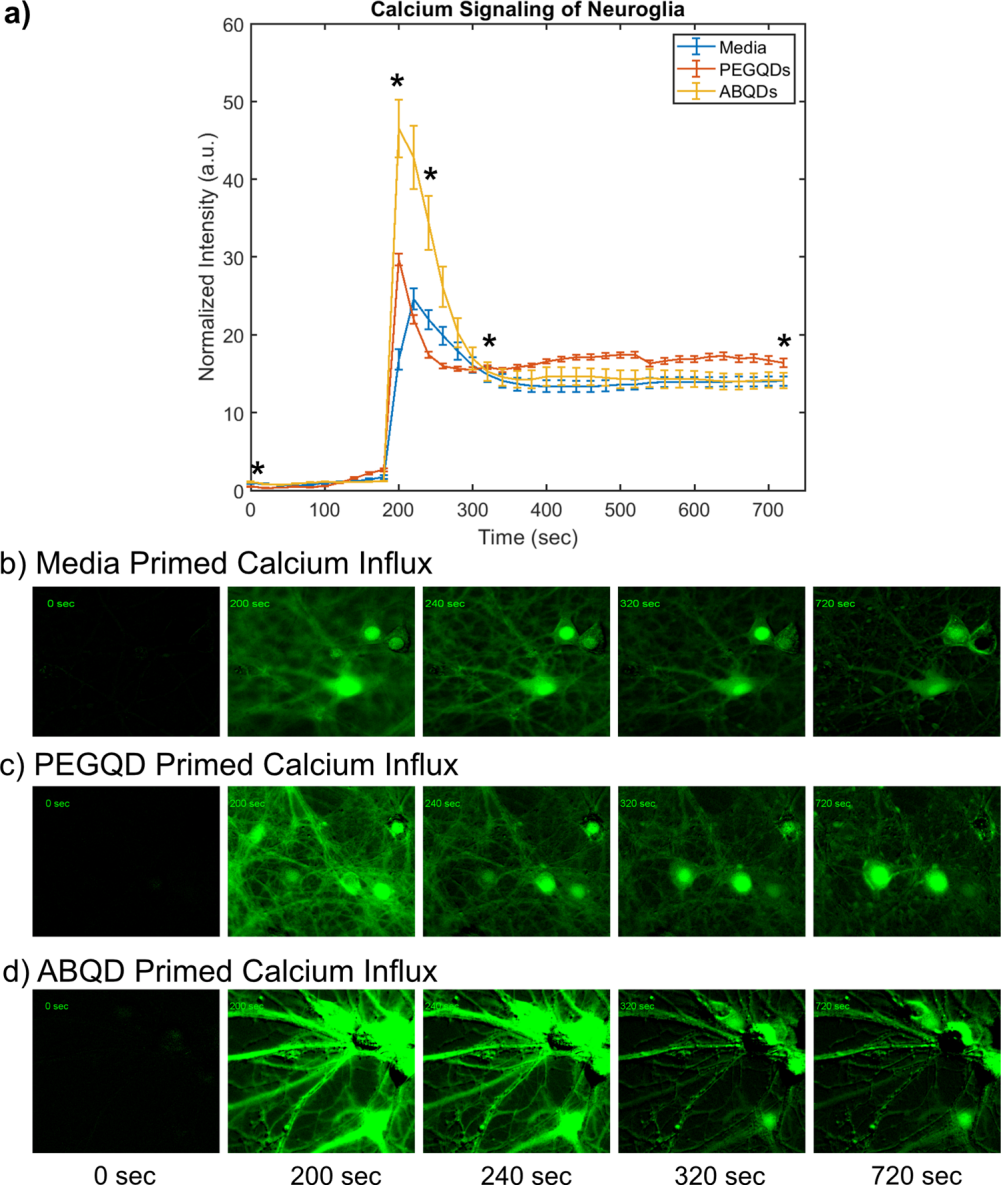
Calcium transients reflecting amyloid-dependent response from neuroglial cultures. (a) Plotted calcium transients arising from cocultures of neurons and astrocytes that were primed with an equal volume dilution of vehicle in media (b), 10 nM of PEGQDs (c), or 10 nM of ABQDs (d) followed by 30 mM KCl calcium induction. Asterisks mark time points that were pulled out for the paneled frames.

Previous studies have linked AβOs to excitotoxicity in neurons due to dysregulation of intracellular calcium transients, leading to the neuroinflammatory hallmarks of synaptic and dendritic damage that we observed in Figure 3.^34,65−67^ To further validate the ability of ABQDs to induce an AβO-associated pathophysiological response in neurons, we examined dysregulation of homeostatic calcium transients in response to the ABQD nano-bio assemblies. Neuronal cultures were treated with an equivalent dilution of nanopure water (negative control; vehicle or veh), 10 nM PEGQDs (negative control), or 10 nM of ABQDs for 10 min, followed by induction of a calcium transient after treatment with 30 mM KCl. As shown in Figure 4, the vehicle and PEGQD treated cultures have no observable difference in the magnitude of calcium transients, as measured by the total fluorescent intensity normalized to the area of interest. In contrast, ABQDs induce a much stronger calcium response (1.5× to PEGQDs and 1.75× to vehicle), observable at the 200 s frame (Figure 4b–d) and the normalized intensity shown in Figure 4a. This increased calcium influx in the ABQD-treated neuronal cultures suggests excitotoxic stress in neurons with observed neurite damage.

### Microglia Exhibit Amyloid-Specific Phagocytic Response to ABQDs

**Figure 5:**
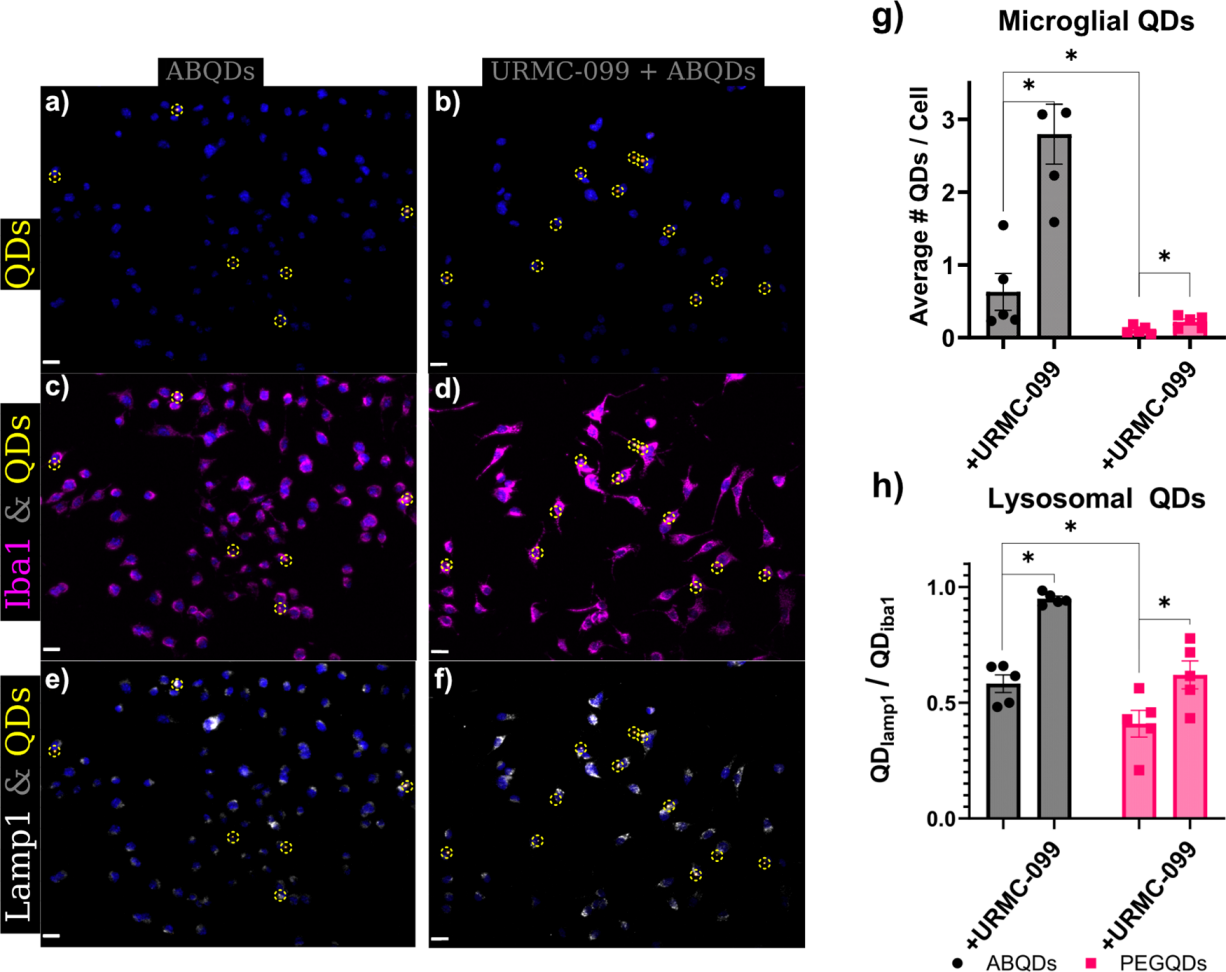
Amyloid-dependent uptake and autophagy of ABQDs. Co-treatment of BV-2 cultures with 100 nM URMC-099 increased the observed ABQD-puncta associated with Iba1+ cells compared to cultures treated with 50 nM ABQDs alone (a–d). This increase of phagocytosis by microglia was observed in both ABQD and PEGQD treated groups, but the raw numbers of microglia associated in 50 nM PEGQD were considerably negligible. The increase in phagocytosis by URMC-099 cotreatment also resulted in creased autophagy, noted by increased colocalization with Lamp1+ regions in the BV-2 (e,f,h). Nuclei are stained with DAPI in blue. Scale bars are 15 μm. (g) All statistical tests shown were performed using two-way ANOVA + Holm-Sidak posthoc, * *p* < 0.05.

Beyond the direct neurotoxic effects of ABQDs on neurons and astrocytes, we characterized the capacity for ABQDs to interact with microglia in an amyloid-dependent manner that occurs in murine models of AD. Specifically, microglia play an essential role in phagocytic uptake and clearing of extracellular amyloid to ameliorate AβO-associated pathology. However, when the amyloid burden is large, microglial processing of AβOs leads to activation of an ER stress state associated with an unfolded protein response (UPR) and a subsequent pro-inflammatory response.^41,68−70^ Thus, we treated an immortalized microglial cell line (BV-2) with either 50 nM PEGQDs (Figure S4) or 50 nM ABQDs (Figure 5) and examined the differential uptake and intracellular processing of these two constructs.

Without the presence of Aβ(1–16) peptides on the surface, the PEGQDs are observed at much lower frequency, defined by the total number of detected QD micelles normalized to the total number of cells (unique DAPI objects), than the ABQDs are in the BV-2 cultures (Figures S8 and 5a), though in both cases all QD PL is observed to be colocalized with Iba-1 labeled microglia. Specifically, in Figures S8 and 5b, we rarely identified neither ABQD nor PEGQD associated fluorescence that was not coincident with Iba-1 associated fluorescence; these are represented by orange spots outlined with dashed yellow circles and magenta objects, respectively, in the fluorescence images.

Like astrocytes, the differential association of PEGQDs and ABQDs to the microglia likely arises from distinct glial phagocytic and processing pathways for extracellular debris and amyloid.^10,41,50,52,68,70−73^ To test this hypothesis, we treated microglial cultures with 100 nM of URMC-099, which has been previously shown to increase amyloid uptake in microglia, but no substantial increase of polymeric nanoparticles in monocyte-derived macrophages.^41,68,72^ Correspondingly, Figure 5b,d demonstrate an increased number of observed puncta associated with QD PL that is highly correlated with Iba-1+ microglia. Normalizing the count of QD micelles by total number of microglia observed (Figure 5e), there is a statistically significant (*p* < 0.05, two-way ANOVA + Holm-Sidak posthoc) increase in ABQD uptake after URMC-099 treatment. While statistical evaluation finds a similarly significant increase in PEGQD phagocytosis, which aligns with the increased autophagic capacity of URMC-099 treated microglia, the raw numbers are essentially negligible in difference; the increase in PEGQDs phagocytosis goes from less than 1 PEGQD phagocytosed per 20 microglia to 1 in 10 microglia, while ABQD phagocytosis starkly increases from ∼1 per microglia to ∼2.5 per microglia Based on these observations, we conclude that the ABQDs are likely recognized in an amyloid-dependent mechanism that leads to differential phagocytic uptake compared to the negative control of surface-bare PEGQDs lacking amyloid N-terminal peptides. Importantly, while glial phagocytosis of monomeric amyloid and some prefibrillar forms of Αβ42 has been previously reported, phagocytosis of the larger, cytotoxic spheroidal AβOs (such as this ABQD biomimetic) has not been clearly documented.^41,68,70,74^

### URMC-099 Primes Microglial Autophagy Response to Alleviate ABQD-Associated Pro-Inflammatory Activation and ER Stress

To further assess microglial recognition of ABQDs in an amyloid-specific process, we examined the intracellular fate of ABQDs compared to PEGQDs after phagocytic uptake. Amyloid-dependent activation of microglia should result in a pro-inflammatory state and subsequent increased phagocytic activity, as well as induction of ER stress via an unfolded protein response (UPR).^42,73−81^ In line with UPR-mediated stress and inflammatory activation, phagocytosis of ABQDs should be ultimately trafficked to either a proteasomal degradation pathway or autophagy-associated lysosomal digestion. Correspondingly, as shown in Figure 5f,h, there is a significant (*p* < 0.05, two-way ANOVA + Holm-Sidak posthoc) increase the number and fraction of ABQDs in Lamp1 immunostained lysosomes, compared to PEGQDs. Additionally, while the PEGQD treated microglia exhibit a non-negligible fraction of lysosomal association (≥50% of observed microglia-associated PEGQDs are also colocalized with Lamp1), the raw count of PEGQDs in lysosomes, and microglia in general, is small, as reflected by the low frequency of microglia-associated PEGQDs (Figure 5e). In line with these observations, we expected that URMC-099 treatment likely shifted ABQD processing toward the pro-autophagic pathway and led to the marked (*p* < 0.05, two-way ANOVA + Holm-Sidak posthoc) increase in association with Lamp1+ lysosomes shown in Figure 5g,h. In support of these findings, previous studies of URMC-099 have characterized increased autophagy in monocyte-derived macrophages, due to increased nuclear translocation of transcription factor EB (TFEB) via a JNK/mTORC1 axis, and have linked this mechanisms as being responsible for increased autophagolysosomal processing of Aβ monomers in microglia.^41,62,68,72^

As confirmation that these phenotypic changes in microglial activity in response to ABQDs is driven by pro-inflammatory activation and ER stress, we isolated RNA from the BV-2 cells and performed reverse transcription quantitative polymerase chain reaction (RT-qPCR) to examine changes in transcriptional activity of C-X-C motif chemokine ligand 10 (CXCL10) and C/EBP homologous protein (CHOP) as well as differential splicing of X-box binding protein 1 (XBP-1). Changes in intracellular CXCL10 can represent inflammatory activation of the microglia, due to a positive feedback pathway with secreted chemokine in these activated phenotypes that can further recruit inflammatory leukocytes.^78−80^ CHOP is a downstream effector of the PERK-eIF2α ER stress transduction pathway and is a transcription factor associated with apoptosis in many neurodegenerative disorders, such as AD.^75,76,82−84^ In a separate, IRE1-dependent, ER stress pathway, IRE1 activation causes a frame shift in the splicing of XBP1, a transcription factor involved in the regulation of other ER regulatory transcripts and proinflammatory cytokine production; changes in the relative fractions of spliced XBP1 (sXBP1) to unspliced XBP1 (usXBP1) would indicate changes in the activation state of the microglia due to this IRE-1 dependent ER stress response. For example, as a transcription factor sXBP1 regulates various biosynthetic pathways necessary to maintain healthy ER function, but excessive ER stress drives it to exacerbate pro-inflammatory signaling associated in AD and other neuroinflammatory diseases.^75−77,83,84^

**Figure 6:**
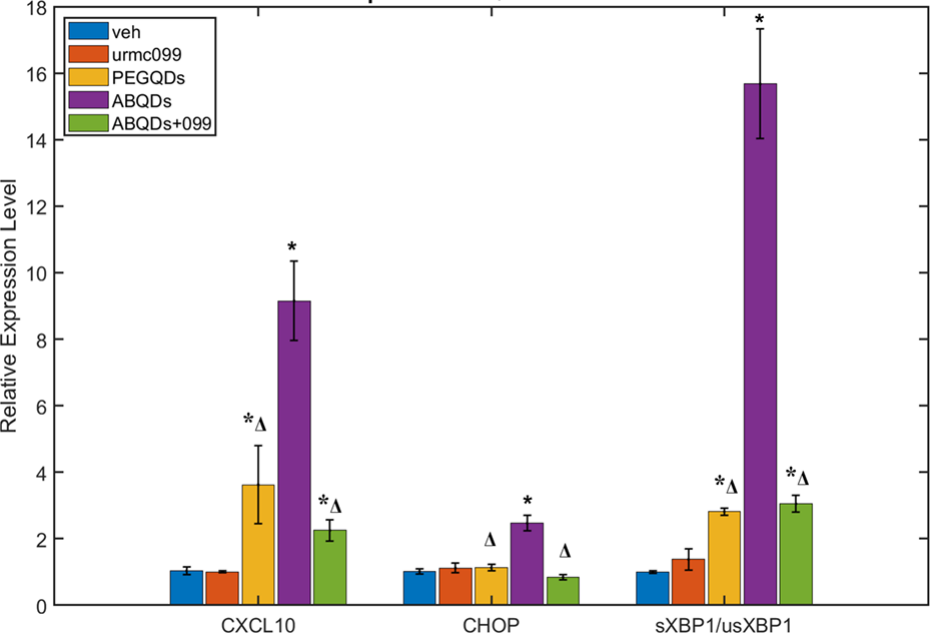
ABQD-mediated ER stress and autophagy signaling in BV-2. CXCL10, CHOP, and splicing variants of XBP1 selected as representative target transcripts for RT-qPCR analysis of inflammatory and ER stress activation specific to ABQDs. * & Δ denote statistically significant differences (*p* < 0.05, one-way ANOVA + Holm-Sidak posthoc) when compared to vehicle or ABQD treatments, respectively.

Changes in CXCL10 transcript levels would give insight into the inflammatory state of the BV-2 cells in various treatment groups, while CHOP transcript levels and changes in sXBP1 to usXBP1 populations would provide insight in ER stress activation, as shown Figure 6. In agreement with our hypothesis, we saw a significant (*p* < 0.05, one-way ANOVA + Holm-Sidak posthoc) increase of CXCL10 and CHOP transcripts due to ABQD treatment as well as increased splicing of XBP1, represented by the fraction of sXBP1 to usXBP1. Furthermore, these effects were ameliorated with URMC-099 treatment, though not to basal levels observed in the two control groups treated with either control (vehicle) or URMC-099 only. This may be attributed to tightly regulated levels of sXBP1 activation and inflammatory activation involved in homeostatic maintenance by microglia.^76−78^ Of interest, the ABQDs seem to act more on the IRE1-XBP1 axis to result in a heightened inflammatory state, as shown by the large increases in sXBP1 and CXCL10, while only a modest, but significant, increase in CHOP. In aggregate, these observations suggest that BV-2 microglia recognize and process ABQDs in an amyloid-associated stress response mechanism that is attenuated with URMC-099 treatment. While URMC-099 has been previously studied in various amyloid and AD models, its capacity to specifically ameliorate damage against larger oligomeric species has not been demonstrated before. Additionally, data from these ABQDs provide potential insight into the specific ER stress pathway activated by the larger spheroidal oligomers that they mimic.

## Conclusions

Understanding the roles of individual oligomeric species of Aβ42 in initiating neuroinflammatory signaling and subsequent pathological events, such as tauopathies, will aid in the precise design of therapeutic strategies to combat AD. In line with this mission, we have designed and constructed a QD biomimetic nano-bio assembly of larger spherical aggregates of AβOs, a proposed cytotoxic initiator of inflammation in AD. The QD core allows this mimetic structure to act as a proxy for an endogenously labeled variant of the native pathologic AβO species, without introducing exogenous labels that may perturb essential aggregation or biomolecular recognition sites. We have validated this ABQD nano-bio assembly to be a structural and functional mimic for the AβOs they closely resemble and have taken advantage of the enhanced optical properties of the QD core to examine its localization in neurons, astrocytes, and microglia. Lastly, the ABQD nano-bio assemblies are used together with URMC-099 to validate the proper activation of inflammatory and stress signaling response pathways associated with AD. This work demonstrates that URMC-099 can facilitate increased phagocytosis and autophagy of amyloid species that are extracellularly aggregated. In future work, we hope to apply this tool to further examine the coordination between AβOs and localized neuronal damage, calcium excitotoxicity, and induction of tauopathies at subcellular resolutions.

## Experimental Methods

For a complete set of materials and methods, please see the associated supplementary document.

### Construction and Characterization of ABQDs

The assembly of ABQDs is comprised of the synthesis of CdSe/CdS QDs followed by encapsulation into a lipid PEG micelle functionalized with Aβ(1–16) peptides.

#### Synthesis of CdSe/CdS

QDs were synthesized using a hot-injection protocol adapted from our previous work to produce CdSe cores, followed by growth of CdS shells.^85−87^ The final oleic acid (OA) capped QDs were suspended in a nonpolar solvent, such as toluene or chloroform and spectrally characterized by absorbance and photoluminescence measurements, complemented by transmission electron microscopy. The QDs are stored in glovebox filled with an inert gas, such as N_2_ until needed.

#### Synthesis of DSPE-PEG_2k_-Aβ

The polymerized phospholipid (DSPE-PEG_2k_-Maleimide) used to form the self-assembled micelles were purchased from Avanti Polar Lipids (cat: 880126C-25 mg). Before micellar encapsulation, the monomers were conjugated with peptides of Aβ(1–16)-PEG-CG via a cysteine-maleimide reaction. The peptides were synthesized via solid-phase peptide synthesis, purified and quantified via reverse phase HPLC, characterized by MALDI-ToF, and lyophilized for storage until needed. The final construct (DSPE-PEG_2k_-Aβ) was purified via dialysis and characterized using analytical HPLC and MALDI-ToF.

#### Assembly of ABQDs

The CdSe/CdS QDs were encapsulated into micelles formed by self-assembly of DSPE-PEG_2k_-Aβ via a modified dual solvent exchange method. In brief, the QDs and lipid-PEG monomers were both suspended in chloroform and then separately sonicated to minimize the presence of preformed aggregates. The solutions were then combined, vortexed and sonicated, then concentrated using a rotary evaporator. As the solution volume approached that of a gel-like film, the solution was removed from the rotary evaporator, nanopure water was added, and then reconnected to the rotary evaporator. The mixture was kept on the rotary evaporator until all organic phase bubbled off and the QDs were transferred into a single aqueous phase. The final product was purified using a 0.1 μm syringe filter, a size exclusion spin filter, and then a round of ultracentrifugation followed by centrifugation of the resuspended pellet. The final pellet was resuspended and both that and the supernatant were analyzed via DLS to determine which fraction to use.

### Primary Neuroglial Cultures

Cell cultures for all experiments consisted of primary mixed rat hippocampal neuronal and astroglia cultures at 18–21 days in vitro (DIV) grown on either glass or fused silica coverslips.

Neuroinflammatory Activation experiments were conducted by exposing the neuroglial cultures to vehicle treatments, 50 nM PEGQDs, 50 nM ABQDs, or 100 nM URMC-099 and 50 nM ABQDs. All treatments were dissolved in neurobasal media (ThermoFisher; cat: 21103049) supplemented with B27 without antioxidants (ThermoFisher; cat: 10889038) and 1% GlutaMax (ThermoFisher; cat: 35050061). Vehicle treatments were either equivolume dilutions of nanopure water or both nanopure and DMSO in the supplemented neurobasal medium. The cultures were incubated at 37 °C and 5% CO_2_ overnight (16–20 h), before fixation and indirect immunofluorescent staining for imaging. Imaging was conducted in a structured illumination format on an Olympus BX51 microscope equipped with an OptiGrid element. The images were processed with Volocity 3D Image Analysis software (PerkinElmer).

*Calcium signaling* was performed by priming the neuroglial cultures with a nanopure vehicle dilution, 10 nM PEGQDs, or 10 nM ABQDs all in supplemented neurobasal media. The priming occurred 10 min, followed by calcium transient induction with a 30 mM KCl solution in supplemented neurobasal medium. The calcium transients were recorded using an inverted microscope with 100 ms exposure times at 20 s intervals over 12 min. The calcium transients were detected using a fluorochrome, Fluo-4AM (ThermoFisher; cat: F14201). The resultant videos were processed using ImageJ-2 (National Institutes of Health).

*Microglia cultures* were comprised of an immortalized murine microglial cell line (BV-2). Before treatment, the cultures were incubated for 1 h in a reduced serum condition comprised of Dulbecco’s Modified Eagle Medium (DMEM, ThermoFisher; cat: 10313039) supplemented with 1% fetal bovine serum (FBS, Atlas Biologicals; cat: F-0500-D), 1% GlutaMax, and 1% penicillin-streptomycin (ThermoFisher; cat: 15140122). This was followed by overnight (16–20 h) incubation at 37 °C and 5% CO_2_ of vehicle, 50 nM PEGQDs, 50 nM ABQDs, or 100 nM URMC-099 with either 50 nM PEGQDs or ABQDs all in the same reduced serum medium. The cells were either then fixed and indirectly immunofluorescent labeled for imaging, or RNA was extracted for RT-qPCR analysis.

## Supporting Information Available

Full experimental procedures, chemical structures, synthetic peptide, and quantum dot characterization data (PDF)
Calcium influx due to vehicle treatment (AVI)
Calcium influx due to PEGQD treatment (AVI)
Calcium influx due to ABQD treatment (AVI)

## Supplementary Material

cn4c00183_si_001.pdf

cn4c00183_si_002.avi

cn4c00183_si_003.avi

cn4c00183_si_004.avi
